# Multiple Links between HD-Zip Proteins and Hormone Networks

**DOI:** 10.3390/ijms19124047

**Published:** 2018-12-14

**Authors:** Giovanna Sessa, Monica Carabelli, Marco Possenti, Giorgio Morelli, Ida Ruberti

**Affiliations:** 1 Institute of Molecular Biology and Pathology, National Research Council, P.le A. Moro 5, 00185 Rome, Italy; giovanna.sessa@uniroma1.it (G.S.); monica.carabelli@uniroma1.it (M.C.); 2 Research Centre for Genomics and Bioinformatics, Council for Agricultural Research and Economics (CREA), Via Ardeatina 546, 00178 Rome, Italy; marco.possenti@crea.gov.it

**Keywords:** Arabidopsis, developmental pathways, environmental responses, HD-Zip transcription factors, hormones

## Abstract

HD-Zip proteins are unique to plants, and contain a homeodomain closely linked to a leucine zipper motif, which are involved in dimerization and DNA binding. Based on homology in the HD-Zip domain, gene structure and the presence of additional motifs, HD-Zips are divided into four families, HD-Zip I–IV. Phylogenetic analysis of *HD-Zip* genes using transcriptomic and genomic datasets from a wide range of plant species indicate that the HD-Zip protein class was already present in green algae. Later, *HD-Zips* experienced multiple duplication events that promoted neo- and sub-functionalizations. HD-Zip proteins are known to control key developmental and environmental responses, and a growing body of evidence indicates a strict link between members of the HD-Zip II and III families and the auxin machineries. Interactions of HD-Zip proteins with other hormones such as brassinolide and cytokinin have also been described. More recent data indicate that members of different HD-Zip families are directly involved in the regulation of abscisic acid (ABA) homeostasis and signaling. Considering the fundamental role of specific HD-Zip proteins in the control of key developmental pathways and in the cross-talk between auxin and cytokinin, a relevant role of these factors in adjusting plant growth and development to changing environment is emerging.

## 1. The HD-Zip Class of Proteins

The homeodomain-leucine zipper (HD-Zip) class of proteins appears to be present exclusively in the plant kingdom and is characterized by the presence of a homeodomain closely linked to a leucine zipper motif [1]. The Arabidopsis genome codes for 48 HD-Zip proteins that, on the basis of sequence homology in the HD-Zip domain, the presence of additional conserved motifs, and specific intron and exon positions, have been grouped into four families: HD-Zip I (17 members), HD-Zip II (10 members), HD-Zip III (5 members) and HD-Zip IV (16 members) [2,3,4,5,6,7]. 

*HD*-*Zip* genes are evolutionary highly conserved and there is evidence that they were already present in green algae [8,9,10,11]. Later in evolution, the HD-Zip class experienced multiple duplication events that promoted neo- and sub-functionalizations for terrestrial life [11].

Experimental work has demonstrated that the HD-Zip domain, but not the HD by itself, interacts with DNA [12], and it has been shown that a correct spatial relationship between the HD and the leucine zipper motif is crucial for DNA binding [12]. Binding-site selection analysis and subsequent chromatin immunoprecipitation sequencing (ChIP-seq) experiments have determined that the HD-Zip proteins recognize pseudo-palindromic DNA elements [3,12,13,14]. HD-Zip I proteins interact with the CAAT(A/T)ATTG motif [12,15,16] whereas HD-Zip II proteins preferentially bind the CAAT(C/G)ATTG motif [12,13]. Binding-site selection analysis identified GTAAT(G/C)ATTAC as the sequence preferentially recognized by HD-Zip III proteins [3]; however, more recent genome-wide binding-site experiments suggest that the AT(G/C)AT central core is sufficient for DNA binding [14]. For HD-Zip IV proteins, the CATT(A/T)AATG motif was shown to be required for DNA binding [13] and found in the promoters of true target genes [17,18,19]. Interestingly, the identified cis-elements are very similar, particularly the HD-Zip II and III binding sites which share the same core sequence [AAT(G/C)ATT] [3,12], thus suggesting that members of the different families of HD-Zip proteins may regulate common target genes [20,21].

**Figure 1:**
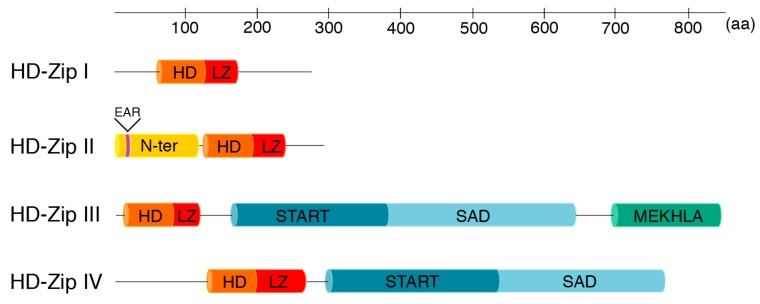
Schematic representation of the protein domains possessed by each HD-Zip family. ATHB1, ATHB2, ATHB8, and GLABRA2 were chosen as representative members of the HD-Zip I, II, III and IV families, respectively. N-term, N-terminus consensus; EAR, LxLxL type of ERF-associated amphiphilic repression; HD, Homeodomain; Zip, Leucine zipper; START, steroidogenic acute regulatory protein-related lipid-transfer domain; SAD, Small body size–mothers against decapentaplegic homolog 4 (Smad4) activation domain; MEKHLA, named after the identification of the highly conserved amino acids Met, Glu, Lys, His, Leu, Ala. Adapted from Ariel et al (2007) [6].

Beside the homeodomain-leucine zipper motif, HD-Zip I proteins have no other established functional domain; conversely, most of the HD-Zip II transcription factors contain an LxLxL type of ERF-associated amphiphilic repression (EAR) motif [7,22] (Figure 1), and there is evidence that they function as negative regulators of gene expression [7,20,23,24,25]. Furthermore, it was recently shown that HOMEOBOX ARABIDOPSIS THALIANA (HAT) 1 and HAT22, two members of the HD-Zip II protein family, interact with the TOPLESS (TPL) co-repressor protein via the EAR motif [26]. In addition to the HD-Zip domain, HD-Zip III and HD-Zip IV proteins contain a steroidogenic acute regulatory protein-related lipid-transfer (START) domain motif with putative lipid-binding capability and a Small body size–mothers against decapentaplegic homolog 4 (Smad4) activation domain (SAD) [27,28] (Figure 1). Finally, HD-Zips III, and not HD-Zips IV, share a MEKHLA domain (Figure 1). A region within this domain contains a region homologous to the PAS (Per-Arnt-Sim)-domain known to act as intracellular sensor of light, oxygen, or redox-potentials [28]. Consistently, it has been reported that REVOLUTA (REV), a member of the HD-Zip III family, acts as a redox-sensitive transcription factor [29]. Furthermore, it has also been shown that the MEKHLA domain is involved in the dimerization of HD-Zip III proteins with DORNROSCHEN (DRN) and DORNROSCHEN-like (DNRL), two APETALA2 (AP2) transcription factors involved in embryo patterning [30].

HD-Zip proteins are known to control key developmental and environmental responses [21,31,32,33]. In particular, a large body of evidence indicates that HD-Zip I and HD-Zip II proteins are involved in environmental responses whereas HD-Zip III and HD-Zip IV proteins act as core developmental factors. However, several recent studies have led to review this conclusion. Indeed, evidence is accumulating that, on one hand, key developmental regulators (members of HD-Zip III and IV families) play an important role in abiotic stress responses and, on the other hand, environmental factors (members of the HD-Zip I and II families) have relevant functions in developmental pathways, thus suggesting that HD-Zip transcription factors may be crucial in adjusting development to changing environment. Here we report recent advances on the understanding of the complex interactions of HD-Zip transcription factors between themselves and with hormone signaling networks, including those involved in abiotic and biotic stress responses.

## 2. HD-Zips I

Sequence comparison and phylogenetic analysis indicated that members of the HD-Zip I protein family can be classified into six different clades, I to VI, and that the presence and the position of conserved sequences may be related to specific function(s) [4,34]. Genome-wide expression studies revealed that several Arabidopsis *HD-Zip I* genes show transcriptional changes in response to treatments with abscisic acid (ABA) [4], and there is evidence that members of clades I and II of the HD-Zip I family have roles related to drought stress and ABA signaling in different plant species [35,36,37,38,39,40,41]. For example, *ARABIDOPSIS THALIANA HOMEOBOX* (*ATHB*) *7* and *ATHB12* (belonging to the clade I) [34] are both strongly induced by water deficit and ABA. Chromatin immunoprecipitation (ChIP) and gene expression analyses have demonstrated that ATHB7 and ATHB12 positively regulate the expression of five genes encoding clade A protein phosphatases type 2C (PP2C), acting as central negative regulators of ABA signaling [42,43,44]. Furthermore, it has also been shown that ATHB7 and ATHB12 act to repress the transcription of two members of the *PYRABACTIN RESISTANCE1* (*PYR1*)/*PYR1-LIKE* (*PYL*) gene family, encoding the ABA receptors [45,46,47]. Together the data indicate that ATHB7 and ATHB12 function as negative regulators of the ABA response in *Arabidopsis* [48]. Evidence exists that HOMEOBOX (HB) 6 (also known as ATHB6, clade II) may also act as a negative regulator of ABA signaling [49]. 

The *HELIANTHUS ANNUUS HOMEOBOX4* (*HAHB4)* gene, encoding a protein homologous to ATHB7 and ATHB12, is also regulated by ABA and drought, as well as by methyl-jasmonic acid (MeJa) or ethylene (ET) or biotic stresses [50,51]. The ectopic expression of HAHB4 in Arabidopsis negatively affects the synthesis of ET and resulted in plants more resistant to drought [50]. In addition, functional analysis of transgenic Arabidopsis and maize plants constitutively expressing HAHB4 suggested that this HD-Zip I protein acts as an integrator of MeJa and ET pathways [51]. 

It is worth mentioning that in some plants there is evidence that ABA synthesis and signaling is relevant to fully activate defense responses against insect herbivores and, in general, hormonal interactions are important for regulating plant responses to abiotic stresses and growth-defense tradeoffs [52]. Of interest is the finding that the *ATHB13* (clade V) gene, positively regulated by low temperature, drought, and salinity, can confer cold, drought and broad-spectrum disease resistance when overexpressed [39,53,54]. The results point to a role of some HD-Zip I proteins as integrators of internal and external signals in the regulation of abiotic and biotic stresses. 

Together with the effects on stresses described above, the overexpression of HD-Zip I proteins very often resulted also in alterations of the shape and growth of the plant, including cotyledon, leaf and supporting organs [55,56], suggesting a role of some HD-Zip I proteins in specific growth and/or developmental pathways. It is worth mentioning that at least *ATHB12* and some ABA signaling components are regulated by KANADI1 (KAN1) [57], a factor controlling organ polarity, including the patterning of leaf primordia in Arabidopsis [58]. Furthermore, evidence strongly suggests that ATHB12 acts as a positive regulator of endoreduplication and cell growth during leaf development [59]. In addition, it has been reported that the *Medicago truncatula HB1*, highly related to *ATHB7* and *ATHB12*, interacts with both ABA and auxin signaling in the regulation of organ development. The *MtHB1* gene is strongly regulated by salt, osmotic and ABA stresses. There is evidence that MtHB1 controls the emergence of lateral roots likely by repressing the auxin-regulated *LOB-Binding Domain 1* (*LBD1*) gene [60], a member of a family of plant-specific transcription factors involved in lateral organ development [61]. 

In the post-embryonic development, ATHB5 (a.k.a. HB5, clade II) behaves as a growth-promoting transcription factor of the hypocotyl. In particular, it promotes the gibberellin acid (GA)-mediated expansion of the epidermal and cortex cells by a positive direct modulation of the expression of *EXPANSIN3* (*EXP3*), a gene involved in cell wall extension [62]. *ATHB1* (Clade III) is a direct target of PHYTOCHROME INTERACTING FACTOR 1 (PIF1), a basic helix-loop-helix (bHLH) transcription factor involved in the regulation of light responses downstream of phytochromes [63] and plays a role in hypocotyl growth under short-day regime likely through a positive regulation of genes involved in cell elongation [64]. Interestingly, *ATHB1* expression is positively regulated by ethylene in Arabidopsis [65], known to regulate the elongation of the hypocotyl in low light and shade [66], whereas the tomato ortholog HB1 directly regulates the expression of *1-AMINOCYCLOPROPANE-1-CARBOXYLATE OXIDASE* (*ACO*) gene encoding a key enzyme in ethylene biosynthesis [67]. 

Very recent work implicated three related HD-Zip I proteins belonging to clade VI [34] in the control of shoot branching [68]. Indeed, it was found that TEOSINTE BRANCHED1, CYCLOIDEA, PCF (TCP) transcription factor BRANCHED1 (BRC1), that functions inside axillary buds to prevent constitutive branch outgrowth [69], binds to and positively regulates the transcription of the *ATHB21*, *ATHB40* and *ATHB53* (a.k.a. *HB21*, *HB40* and *HB53*) genes, all belonging to clade VI. These HD-Zip I proteins are necessary and sufficient to enhance the expression of *9-CIS-EPOXICAROTENOID DIOXIGENASE 3 (NCED3)*, a key ABA biosynthesis gene, and for ABA accumulation inside axillary buds in conditions of low Red/Far-Red (R/FR) ratio light or short photoperiod. This, in turn, causes suppression of bud development. Relevantly, the BRC1/ATHB21/40/53 regulatory module appears to be conserved in monocot and dicot species [69].

Besides the multiple links found between HD-Zip I proteins and ABA, evidence of a direct interaction between HD-Zips I and auxin also exists [70]. Auxin has a central role during embryogenesis and post-embryonic development. The transcriptional auxin response is regulated by AUXIN RESPONSE FACTOR (ARF) transcription factors and AUXIN/INDOLE-3-ACETIC ACID (AUX/IAA) proteins. In the absence of auxin, AUX/IAAs act as repressors by forming heterodimers with ARFs; the auxin-mediated degradation of AUX/IAAs releases the inhibition on ARF transcription factors [71,72]. MONOPTEROS (MP)/ARF5 and its interacting AUX/IAA partner BODENLOS (BDL)/IAA12 play an important role during the embryonic and post-embryonic development. Both a dominant mutant of *(BDL)/IAA12* and a loss-of-function mutant of *MP/ARF5* lack a seedling root and display cotyledon defects [73,74,75,76]. Interestingly, it was found that the ATHB5 protein (clade II) directly negatively regulates *BDL/IAA12* expression. Overexpression of ATHB5 during embryogenesis transcriptionally suppresses the expression of *BDL/IAA12* and rescues the rootless phenotype of the *bdl/iaa12* dominant mutant. Together the data lead to the hypothesis that ATHB5 may contribute to spatially restrict *BDL/IAA12* expression during embryogenesis [70]. Evidence that ATHB6, a close homolog of ATHB5, may act redundantly with ATHB5 in the negative regulation of *BDL/IAA12* have also been provided [70]. 

Finally, the cross-talk between ethylene and auxin in the control of root elongation mediated by ATHB52 has been recently uncovered. It is very well established that root elongation is inhibited by ethylene in Arabidopsis and other species through the action of auxin [77,78,79,80]. The *ATHB52* gene is positively regulated by ETHYLENE-INSENSITIVE3 (EIN3), a key transcription factor of the ethylene signal transduction pathway. A molecular and genetic analysis has shown that ATHB52 binds the promoters of *PIN FORMED2 (PIN2)*, coding for a polar auxin carrier, and of *WAVY ROOT GROWTH1* (*WAG1*) and *WAG2*, encoding PIN polarity regulators. The positive modulation by ethylene of the PIN2/WAG1/WAG2 module exerted through ATHB52 could affect the local polar auxin transport in the root tip resulting in the inhibition of primary root elongation [81].

## 3. HD-Zips II

The HD-Zip II protein family contains ten members which can be divided into four clades (α-δ) [7]. Remarkably, all the *HD-Zip II γ (ATHB2, HAT1, HAT2)* and *δ* (*HAT3*, *ATHB4*) genes are rapidly induced by changes in the R/FR ratio light that promote shade avoidance in the Angiosperms [7,82] and several evidence exist that HD-Zip II γ and δ proteins act as positive regulators of this response [7,23,25,83,84,85,86,87].

Interestingly, evidence demonstrates that, besides their function in shade avoidance, HD-Zip II γ and δ transcription factors play a crucial role in embryo apical development and essential developmental processes in sunlight, including shoot apical meristem (SAM) activity, organ polarity and gynoecium development [20,31,88,89,90,91].

Several links have been established between HD-Zip II γ and δ proteins and auxin. Plants with elevated levels of ATHB2 display a constitutive shade avoidance response, and it was shown that ATHB2-induced elongation of the hypocotyl depends on the auxin transport system, as it is abolished by auxin transport inhibitors [23]. Furthermore, the lateral root phenotype observed in ATHB2 overexpressing seedlings is rescued by IAA [23]. Finally, it was found that both auxin synthesis and transport are affected in *hat3 athb4* and *hat3 athb4 athb2* mutant embryos [20,31]. 

**Figure 2:**
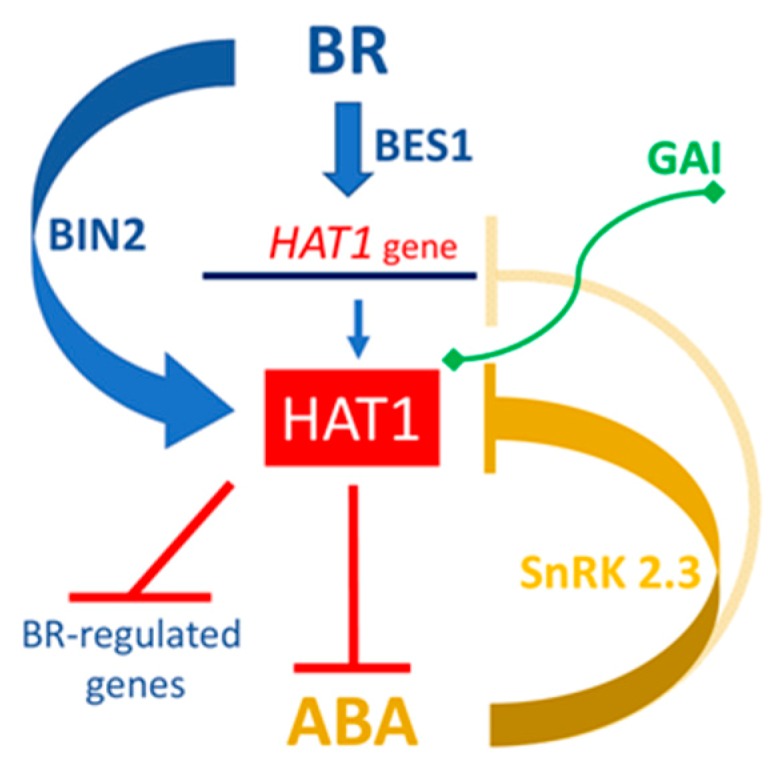
Positive and negative hormonal pathways regulated by HAT1. BR positively regulates the expression of *HAT1* through the BES1 transcription factor and stabilizes HAT1 protein through the BIN2 kinase. HAT1 acts together with BES1 as a transcriptional repressor of BR-regulated genes. HAT1, whose expression is negatively regulated by ABA at the transcriptional and post-transcriptional levels, represses the expression of *NCED3* and *ABA3* resulting in a reduction of ABA synthesis. The SnRK 2.3 kinase, positively acting in the ABA signaling, affects both HAT1 protein stability and DNA binding activity. The GAI interaction with HAT1 is also indicated.

Recently, HAT1 has been linked to brassinosteroid (BR) signaling pathway. BRs signal through a plasma membrane-localized receptor kinase to modulate the BES1/BZR1 (BRI1-EMS SUPPRESSOR 1/BRASSINAZOLE RESISTANT1) family of transcription factors that positively and negatively regulate a large number of genes [92,93,94,95,96,97]. Relevantly, it was recently found through ChIP experiments that *HAT1* is a direct target of BES1 [98]. HAT1 functions redundantly with its close homolog HAT3, as the double loss-of-function mutant *hat1 hat3* displayed a reduced BR response stronger than that of the *hat1* and *hat3* single mutants. Expression levels of several BR-repressed genes are increased in *hat1 hat3* double mutant and reduced in HAT1 overexpressing lines, thus strongly suggesting that HAT1 functions to repress the expression of a subset of BR target genes. Consistently, it was found that HAT1 binds to DNA elements in BR-repressed gene promoters and functions as a BES1 corepressor [98]. Furthermore, it was shown that GSK3 (GLYCOGEN SYNTHASE KINASE 3)-like kinase BIN2 (BRASSINOSTEROID-INSENSITIVE 2), a negative regulator of the BR pathway, increases the stability of HAT1 [98,99,100,101] (Figure 2). 

Furthermore, HAT1 was identified among the transcription factors interacting with the GIBBERELLIN INSENSITIVE (GAI) DELLA protein, a master negative regulator in gibberellin (GA) signaling [102,103] (Figure 2). Further work is needed to establish the specific GA-response(s) in which HAT1 is involved.

Recent work has demonstrated that HAT1, apart from its role in BR-mediated growth responses [98], in GA signaling, and in viral defense response in a manner dependent on salicylic acid (SA) [104], it also negatively regulates, redundantly with HAT3, ABA-mediated drought responses through suppression of ABA biosynthesis and signaling [105]. The expression of both *HAT1* and *HAT3* is indeed repressed by ABA. Evidence have been provided that HAT1 can bind to specific DNA sequences on the promoters of *NCED3* and *ABA DEFICIENT* (*ABA*) *3*, two key ABA biosynthesis genes, and negatively regulate their expression, thus resulting in a reduction of ABA synthesis. In addition, it was observed that HAT1 overexpressing plants display reduced sensitivity to ABA and less tolerance to drought stress, whereas the double loss-of-function *hat1 hat3* mutant show opposite phenotypes. Finally, it was found that Sucrose non-fermenting 1-related protein kinase (SnRK) 2.3, a positive component of ABA signaling, physically interacts with and phosphorylates HAT1, decreasing its protein stability and binding activity [105] (Figure 2).

Relevantly, at least other two HD-Zip II proteins, ATHB17 and HAT22/ABA-INSENSITIVE GROWTH 1 (ABIG1), are linked to ABA [106,107]. *ATHB17* expression is induced by ABA, and evidence have been provided that *athb17* loss-of-function mutants are ABA-insensitive and drought-sensitive whereas lines overexpressing ATHB17 display opposite phenotypes. Interestingly, the effect of ATHB17 on seedling growth in the presence of ABA is stage-specific. Indeed, it is observed exclusively during the post-germination seedling establishment stage [106]. Recent work identified HAT22/ABIG1 as a transcription factor required for ABA-mediated growth inhibition, but not for seed dormancy and stomatal closure. It has been proposed that drought acts through ABA to increase *HAT22/ABIG1* transcription which, in turn, inhibits new shoot growth and promotes leaf senescence [107].

## 4. HD-Zips III

The HD-Zip III family contains five members: ATHB8, CORONA (CNA), PHABULOSA (PHB), PHAVOLUTA (PHV), and REV. It is well established that HD-Zip III proteins act as master regulators of embryonic apical fate [108], are required to maintain an active SAM and to establish lateral organ polarity [109,110,111] and are necessary for xylem formation and specification [112,113,114,115,116,117]. Recent work has also implicated HD-Zip III proteins in the regulation of the shade avoidance response [14,21,32].

The pattern of HD-Zip III expression largely coincides with that of auxin distribution [8,9,115,118,119,120,121,122]. Furthermore, *HD-Zip III* genes are regulated at the post-transcriptional level by the microRNAs miR165/166, which negatively affect their expression through mRNA cleavage [110,123]. Relevantly, REV directly positively regulates the *HD-Zip II* genes *HAT3*, *ATHB4*, *ATHB2*, and *HAT2*, and evidence exists that PHB and PHV are involved in the control of *HAT3* expression [14,20]. It has been recently shown that REV physically interacts with HAT3 and ATHB4 to directly repress MIR165/166 expression in the adaxial side of the leaf [91].

The interconnection between HD-Zip III transcription factors and auxin began to be clarified especially through the molecular-genetic analysis of the vascular system [124]. REV directly positively regulates the auxin biosynthetic genes *TRYPTOPHAN AMINOTRANSFERASE OF ARABIDOPSIS 1* (*TAA1*) and *YUCCA5* (*YUC5*) [14,125]. Furthermore, it has been demonstrated that genes implicated in auxin transport, including the influx carriers *LIKE AUXIN RESISTANT 2* (*LAX2*) and *LAX3*, and response are also direct targets of REV [122,125,126]. Interestingly, REV also upregulates the expression of *NAKED PINS IN YUC MUTANTS 1* (*NPY1*) and *WAG1*, encoding an AGC protein kinase highly similar to PINOID (PID) [127]. *NPY* genes encode proteins with a Broad-Complex, Tramtrack, and Bric-a-brac (BTB)-Poxvirus and Zinc Finger (POZ) domain that together with AGC kinases determine the subcellular polar targeting of the PIN efflux carriers, thus establishing the direction of auxin transport [128,129,130,131].

A recent study reinforces the interconnection between HD-Zip III transcription factors and auxin signaling [132]. HD-Zip III proteins are known to determine xylem patterning in the Arabidopsis root [116], and it has been shown that PHB directly interacts with the promoter of both *MP/ARF5*, a transcription factor gene playing a major role in vascular development, and *IAA20*, encoding an IAA protein that is stable in the presence of auxin and able to interact with MP. The double mutant of *IAA20* and its closest homolog *IAA30* forms ectopic protoxylem, whereas elevated levels of IAA30 result in discontinuous protoxylem, analogous to a weak *mp* mutant. It has therefore proposed a mechanism in which PHB stabilizes the auxin response within the xylem axis by activating both MP and its repressors IAA20 and IAA30 to ensure correct vascular patterning and differentiation of xylem cells [132].

Cross-talk between auxin and cytokinin (CK) is crucial during several developmental processes, including vascular development. Several studies have indicated that ARABIDOPSIS HISTIDINE PHOSPHOTRANSFER PROTEIN 6 (AHP6), an inhibitory pseudophosphotransfer protein, is positively regulated by auxin and counteracts CK signaling, allowing protoxylem formation in the root. Conversely, CK signaling negatively regulates the spatial domain of AHP6 expression [133,134]. Interestingly, it has been shown that PHB acts redundantly with other HD-Zip III transcription factors to downregulate *AHP6* expression, either directly or by alteration of auxin signaling [116,133].

**Figure 3:**
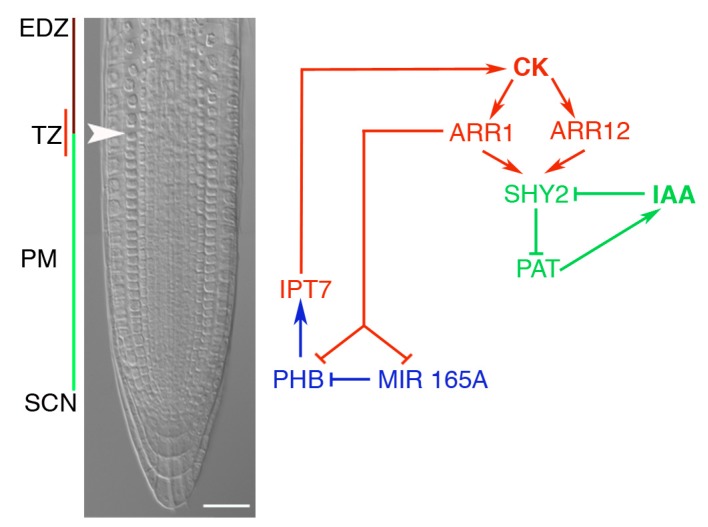
Interplay between the HD-Zip III protein PHB and CKs in the regulation of root meristem size. DIC image of the Arabidopsis root tip with different developmental zones indicated, stem cell niche (SCN), proximal meristem (PM), transition zone (TZ), and elongation and differentiation zone (EDZ), together with a schematic representation of the molecular interactions involved in the determination of RAM size. PHB induces CK biosynthesis in the PM of the root; cytokinin is delivered to the transition zone where activates ARABIDOPSIS RESPONSE REGULATOR (ARR) 1. ARR1 represses the expression of PHB at the TZ, thus restricting PHB expression to the distal part of the PM. Remarkably, ARR1 also represses the transcription of *MIR165A* [135]. In addition, ARR1 induces the expression of *SHY2*, a CK primary target necessary and sufficient to promote cell differentiation at the TZ. White arrowhead indicates the TZ of the cortex tissue, placed at the boundary between the last meristematic cell and the first differentiating cell [137]. A color code has been used to indicate different pathways: CK pathway, red; IAA pathway, green; PHB/miR165, blue. Scale bar, 20 μm.

The interaction between HD-Zip III proteins and cytokinin network is further strengthened by the finding that PHB directly activates *ISOPENTENYLTRANSFERASE 7* (*IPT7*), a gene coding for a rate-limiting component of the cytokinin biosynthesis pathway. This in turn promotes cell differentiation and regulates root length [135]. These results, together with the finding that CK is transported in the phloem [136], suggests that CK is synthesized in the meristem vasculature through the activity of PHB and then delivered to the transition zone (TZ) to promote differentiation. Consistently, the expression of *SHORT HYPOCOTYL 2* (*SHY2*), a CK primary target necessary and sufficient to promote cell differentiation at the TZ [137], is weaker in *phb phv* double loss-of-function mutants with respect to wild type but is reestablished after CK treatment. In addition, the root meristem phenotype of the dominant *shy2-2* mutant is suppressed in *phb phv*, further confirming the hypothesis that PHB-dependent CK biosynthesis in the distal part of the root influences cell differentiation at the proximal TZ [135]. Relevantly, it was also shown that CK represses both *PHB* and *miR165* [135]. The authors referred to these interactions as an incoherent regulatory loop in which CK represses both its activator and a repressor of its activator and proposed that this circuit might provide robustness against CK fluctuations [135] (Figure 3).

Furthermore, recent work has shown that PHB also regulates cell activities at the TZ by repressing B-ARRs, positive regulators of CK signaling [138,139]. Such a repressive effect of PHB on B-ARR activities is enhanced by high cytokinin [139].

A recent study on the mechanism underlying shoot regeneration in Arabidopsis provided an additional link between HD-Zip III transcription factors and CK signaling [140], somehow predicted also on the basis of protein-protein interaction studies between HD-Zip III and DNR/DNRL transcription factors [30,141,142] and the analysis of the regeneration capacity of a *cna* mutant encoded by the *hoc* locus [143]. Four B-ARR transcription factors, ARR1, ARR2, ARR10, and ARR12, have essential roles in shoot regeneration. Indeed, the shoot regenerative capacity is impaired in the *arr1 arr10 arr12* mutant with respect to wild type [144,145,146]. The A-type ARRs play opposite roles in shoot regeneration, and it has been shown that overexpression of ARR7 or ARR15 results in a marked reduction of the regeneration capacity [147]. Remarkably, it was found that ARR1, ARR2, ARR110 and ARR12 interact with PHB, PHV and REV HD-Zip III transcription factors, and that these complexes in turn activates the expression of *WUSCHEL* (*WUS*), a gene essential in maintaining SAM activity [140,148].

Beside the evidence of molecular interactions between HD-Zip III proteins and key components of the auxin and cytokinin networks, it has been recently reported that REV positively directly regulates the expression of the gene encoding the ABA receptor protein PYL6 [125,126]. Remarkably, *PYL6* is oppositely directly regulated by KAN1, a key determinant of abaxial cell fate in the leaf [59,127,149]. Furthermore, microarray data revealed that the expression of *REV*, *PHB* and *PHV* significantly decreases upon ABA application, as a consequence of ectopic induction of miR165 expression [21,126]. It has been therefore proposed that the connection between ABA perception and signaling and HD-Zip III transcription factors may be required to adapt leaf development to alterations in water availability [21,126].

Finally, it is worth mentioning that recent work has revealed that the expression of miR165/166 is regulated by a complex hormonal cross-talk during root development in Arabidopsis [150]. Evidence have been provided that miR 165/166 has important functions in ABA and abiotic stress responses. It was indeed found that reduction in the expression of miR165/166 results in drought and cold resistance phenotypes and hypersensitivity to ABA during seed germination and seedling development. Furthermore, it was shown that miR165/166-mediated regulatory module is linked with ABA responses likely through a direct regulation by HD-Zip III proteins of ABA INSENSITIVE4 (ABI4), a regulator of ABA signaling, and β-glucosidase 1 (BG1), known to hydrolyze glucose-conjugated, biologically inactive ABA to produce active ABA [151]. In addition, there is also evidence that the cross-talk between miR165 and ABA is involved in root xylem formation and vascular acclimation to water deficit. It was indeed shown that under limited water availability endodermal ABA signaling activates *MIR165A*, thus leading to increased miR165 levels that repress HD-Zip III transcription factors in the stele. Together the data nicely show how a pathway known to control core developmental processes is used as a mean to adjust xylem formation under conditions of abiotic stress [152].

## 5. HD-Zips IV

Many of the *HD-Zip IV* genes were shown to be specifically or preferentially expressed in the epidermis of developing embryos and/or other plant organs [153]. It is worth mentioning that the epidermis plays a critical role also in plant defense against pathogens and in protection from environmental stresses [154,155].

GLABRA2 (GL2), the first identified member of the HD-Zip IV protein family, promotes trichome differentiation [156], and suppresses root hair formation in the root epidermis [19,156]. In particular, GL2 controls cell fate determination of N-cells (non-hair cells; atrichoblast) [157], through a negative regulation of the phospholipase D zeta 1 (PLDz1) [18], and of several bHLH transcription factors including ROOT HAIR DEFECTIVE 6 (RDH6) involved in root hair initiation [156]. There is strong evidence that BRs are required to maintain position-dependent cell fate specification in root epidermis, as loss of BR signaling results in loss of H-cells (hair cells; trichoblast). Indeed, BRs are required for a correct expression of *WEREWOLF* (*WER*) and *GL2*, master regulators of epidermal patterning, and of *CAPRICE* (*CPC*), a direct downstream target of WER [158].

Recent work has also established a link between HD-Zip IV proteins and GA signaling [159]. It was indeed shown that DELLA proteins interact directly with MERISTEM LAYER 1 (ATML1) and its paralogue PLANT DEFENSIN 2 (PDF2), two HD-Zip IV proteins required for epidermis specification and binding to the L1 box present in the promoters of epidermis-specific genes [17,160]. Silencing of both ATML1 and PDF2 inhibits epidermis-specific gene expression and delays germination [157]. Evidence were provided that, upon seed imbibition, increased GA levels reduce DELLA protein levels, thus releasing ATML1/PDF2 to activate epidermis-specific expression and promote seed germination [159]. 

Apart from their role in development, interactions between HD-Zip IV transcription factors and environmental responses have also been reported [161]. For example, *HOMEODOMAIN GLABRA 11* (*HDG11*) was identified via activation tagging as a gene involved in drought tolerance. The mutant has higher levels of ABA than the wild type and displays enhanced root growth with more lateral roots and reduced stomatal density. Overexpression of HDG11 also conferred drought tolerance associated with augmented lateral roots and reduced leaf stomatal density in both Arabidopsis and tobacco [162]. Higher level of ABA and improved drought tolerance have been observed also in cotton, poplar and rice transgenic plants overexpressing HDG11 [161,163]. It has been suggested that HDG11 positively regulates the expression of cell-wall-loosening protein genes, including *EXP5,* resulting in a well-developed root system [164].

Intriguingly, HDG11 acts as a maternal regulator of zygote asymmetry through a direct activation of the *WUSCHEL RELATED HOMEOBOX 8* (WOX8) gene whose product leads to asymmetric division of the zygote [165]. 

## 6. Conclusions

A fundamental question in plant biology is how plants integrate environmental signals with intrinsic developmental programs and how coordinate the growth of different organs depending on resource availability. Over recent years remarkable progress has been made, and the molecular mechanisms controlling these processes are being elucidated. The functional analysis of the HD-Zip proteins revealed that they are part of complex networks involved in the integration of external signals through the regulation of hormonal pathways involved in the control of fundamental developmental processes. Although many factors belonging to each HD-Zip family are implicated in specific processes, it is interesting to note that the simple overexpression of ATHB2 (and other members of the HD-Zip II family) and HDG11 is sufficient to generate plants looking for optimal light and water for growth, respectively. This could be explained by the existence of organ- and/or tissue-specific hubs that stimulated by external and/or internal (hormonal) signals converge to coherently adjust the development and growth to a specific environmental signal. These hubs may have been generated during evolution through multiple duplication events that have promoted neo and sub-functionalization of factors operating on specific pathways. The identification of these hubs it has the potential to lead to a more unified vision of the development and growth of the plant according to environmental stresses that could be applied for the improvement of the cultivated plants.

## Abbreviations

ABA	Abscisic acid
ABA	ABA deficient
ABI4	ABA INSENSITIVE4
ABIG1	ABA-INSENSITIVE GROWTH 1
ACCO	1-AMINOCYCLOPROPANE-1-CARBOXYLATE OXIDASE
AHP6	HISTIDINE PHOSPHOTRANSFER PROTEIN 6
AP2	APETALA2
AUX/IAA	AUXIN/INDOLE-3-ACETIC ACID
ARF	AUXIN RESPONSE FACTOR
ARR	ARABIDOPSIS RESPONSE REGULATOR
ATHB	ARABIDOPSIS THALIANA HOMEOBOX
ATML1	MERISTEM LAYER 1
BDL	BODENLOS
BES1/BZR1	BRI1-EMS SUPPRESSOR 1/BRASSINAZOLE RESISTANT1
BG1	β-glucosidase 1
b-HLH	basic Helix-Loop-Helix
BIN2	BRASSINOSTEROID-INSENSITIVE 2
BR	Brassinosteroid
BRC1	BRANCHED1
ChIP	Chromatin Immunoprecipitation
ChIP-seq	Chromatin Immunoprecipitation sequencing
CK	Cytokinin
CNA	CORONA
CPC	CAPRICE
DRN	DORNROSCHEN
DNRL	DORNROSCHEN-like
EAR	ERF-Associated Amphiphilic Repression
EDZ	Elongation and Differentiation Zone
EIN3	ETHYLENE-INSENSITIVE 3
EXP3	EXPANSIN 3
ET	Ethylene
H cell	Hair cell; trichoblast
HAHB4	HELIANTHUS ANNUUS HOMEOBOX 4
HB	HOMEOBOX
HAT1	HOMEOBOX ARABIDOPSIS THALIANA 1
HDG11	HOMEODOMAIN GLABRA 11
HD-Zip	Homeodomain-leucine zipper
IPT7	ISOPENTENYLTRANSFERASE 7
GA	Gibberellin Acid
GAI	GIBBERELLIN INSENSITIVE
GL2	GLABRA2
GSK3	GLYCOGEN SYNTHASE KINASE 3
KAN1	KANADI 1
LAX2	LIKE AUXIN RESISTANT 2
LBD1	LOB-Binding Domain 1
MeJa	Methyl-Jasmonic acid
MP	MONOPTEROS
N cell	Non-hair cell; atrichoblast
NCED3	9-CIS-EPOXICAROTENOID DIOXIGENASE 3
NPY1	NAKED PINS IN YUC MUTANTS 1
PDF2	PLANT DEFENSIN 2
PHB	PHABULOSA
PHV	PHAVOLUTA
PID	PINOID
PIF1	PHYTOCHROME INTERACTING FACTOR 1
PIN2	PIN FORMED2
PLDz1	Phospholipase D zeta 1
PM	Proximal Meristem
POZ	Broad-Complex, Tramtrack, and Bric-a-brac (BTB)-Poxvirus and Zinc Finger
PP2C	Protein Phosphatases type 2C
PYL	PYRABACTIN RESISTANCE1 (PYR1)/PYR1-LIKE
RDH6	ROOT HAIR DEFECTIVE 6
R/FR	Red/Far-Red
REV	REVOLUTA
SAD	Small body size–mothers against decapentaplegic homolog 4 (Smad4) activation domain
SAM	Shoot Apical Meristem
SCN	Stem Cell Niche
SHY2	SHORT HYPOCOTYL 2
SnRK	Sucrose non-fermenting 1-related protein kinase
START	Steroidogenic acute regulatory protein-related lipid-transfer
TAA1	TRYPTOPHAN AMINOTRANSFERASE OF ARABIDOPSIS 1
TCP	TEOSINTE BRANCHED1, CYCLOIDEA, PCF
TPL	TOPLESS
TZ	Transition Zone
WAG1	WAVY ROOT GROWTH 1
WER	WEREWOLF
WOX8	WUSCHEL RELATED HOMEOBOX 8
WUS	WUSCHEL
YUC5	YUCCA5
